# Enhancing clinical potential of liquid biopsy through a multi-omic approach: A systematic review

**DOI:** 10.3389/fgene.2023.1152470

**Published:** 2023-04-03

**Authors:** Gianna Di Sario, Valeria Rossella, Elvira Smeralda Famulari, Aurora Maurizio, Dejan Lazarevic, Francesca Giannese, Claudia Felici

**Affiliations:** Centre for Omics Sciences, IRCCS San Raffaele Scientific Institute, Milan, Italy

**Keywords:** personalized medicine, tumor biomarker, circulating tumor cell (CTC), circulating tumor DNA (ctDNA), multi-omics, exosome, miRNA, liquid biopsy

## Abstract

In the last years, liquid biopsy gained increasing clinical relevance for detecting and monitoring several cancer types, being minimally invasive, highly informative and replicable over time. This revolutionary approach can be complementary and may, in the future, replace tissue biopsy, which is still considered the gold standard for cancer diagnosis. “Classical” tissue biopsy is invasive, often cannot provide sufficient bioptic material for advanced screening, and can provide isolated information about disease evolution and heterogeneity. Recent literature highlighted how liquid biopsy is informative of proteomic, genomic, epigenetic, and metabolic alterations. These biomarkers can be detected and investigated using single-omic and, recently, in combination through multi-omic approaches. This review will provide an overview of the most suitable techniques to thoroughly characterize tumor biomarkers and their potential clinical applications, highlighting the importance of an integrated multi-omic, multi-analyte approach. Personalized medical investigations will soon allow patients to receive predictable prognostic evaluations, early disease diagnosis, and subsequent *ad hoc* treatments.

## Introduction

In the last decades, the old “one-size-fits-all” approach in cancer treatment has been replaced by a personalized model in which therapeutic strategy is based on biological features of the patient’s disease (Gambardella et al., 2020; Kulavi et al., 2021). This approach, known as personalized medicine, aims to identify patients who will respond to specific therapies by reducing the risk of adverse effects as well as improving the sustainability of healthcare systems (Biankin et al., 2015; Jameson and Longo, 2015; Hyman et al., 2017; Yates et al., 2018). During the selection of “the right treatment for the right person,” the identification of a new generation of biomarkers, that guide all aspects of cancer patient care, represents the most urgent challenge today (Sawyers, 2008). Tissue-based biomarkers are currently used for tumor diagnosis and therapy response prediction; however, they do not allow treatment real-time monitoring and early identification of resistance mechanisms. In some cases, tissue biopsy is invasive, expensive and time consuming, but above all, it is totally inappropriate in capturing tumor heterogeneity. Indeed, we can identify only a fraction of tumor heterogeneity since serial sampling is not clinically practical and is affected by the patient’s health status.

**FIGURE 1:**
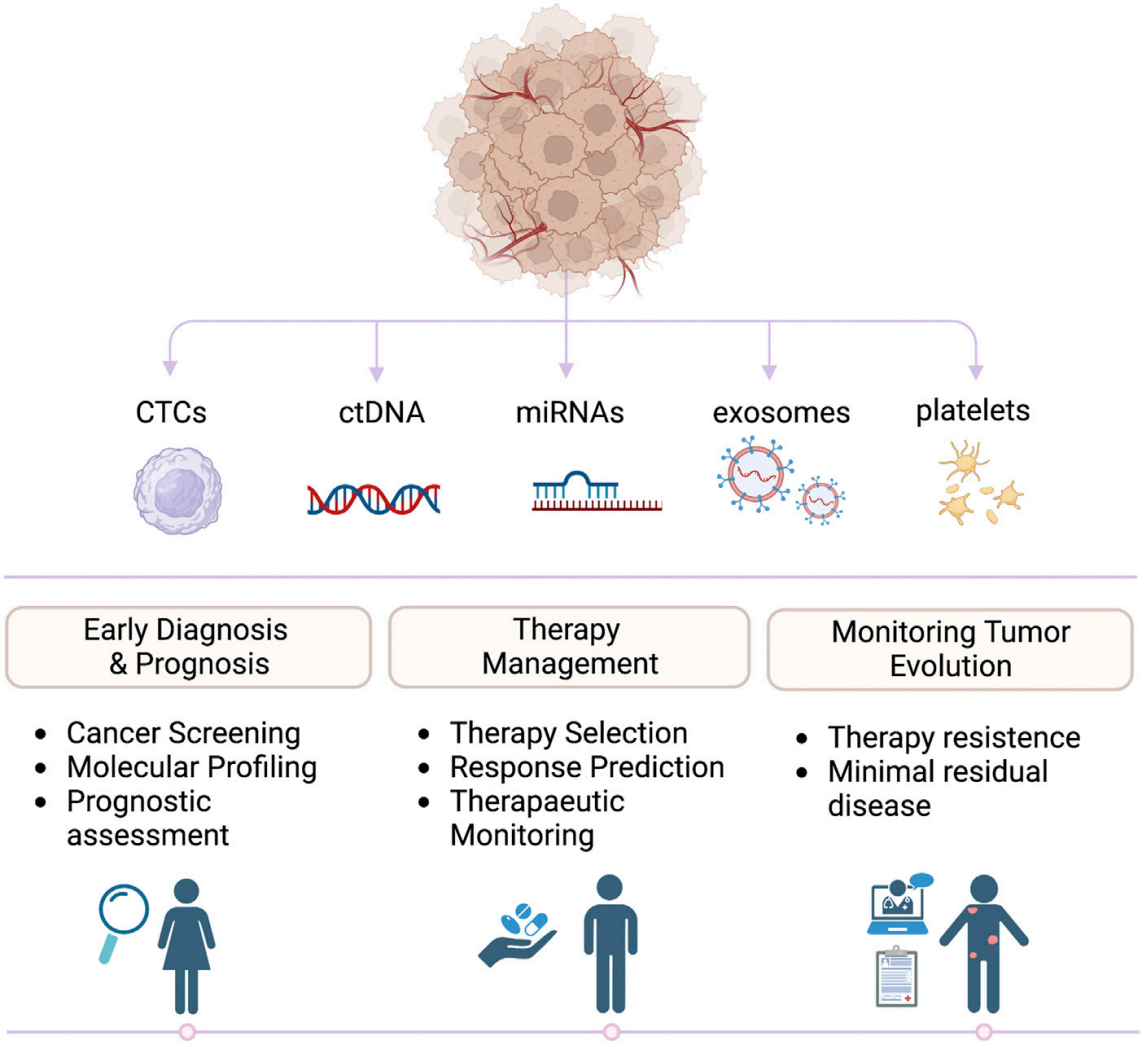
Clinical applications of liquid biopsies. Biomarkers released by primary tumors and metastasis can be detected in liquid biopsies and analyzed to guide prognosis, diagnosis, and treatments in oncologic patients.

To overcome these limitations, a non-invasive sampling approach known as liquid biopsy (LB) was developed (Lone et al., 2022). Based on the study of circulating biomarkers in biological fluids, it aims to circumvent the temporal and spatial heterogeneity of the tumor by providing valuable information on the onset and progression of the disease over time (Siravegna et al., 2017; Ignatiadis et al., 2021). Besides blood, different biofluids such as urine (Nuzzo et al., 2020; Xu et al., 2020), stool (Diehl et al., 2008a), saliva/sputum (Wu et al., 2019), pleural effusions (Li et al., 2019a), and cerebrospinal fluid (CSF) (Miller et al., 2019) can be used to isolate circulating tumor components for clinical applications (Figure 1). Biologically, elements that can be analyzed using LB are classified into two main categories: targets with cellular or subcellular structures such as circulating tumor cells (CTCs) (Zhang et al., 2015; Jordan et al., 2016; Guo et al., 2018; Zhou et al., 2019), extracellular vesicles (EVs) (McKiernan et al., 2016; Mannavola et al., 2019; Sun et al., 2020) and tumor-educated platelets (TEPs) (Best et al., 2015); and molecules without cellular structures such as cell-free (cf) nucleic acids (cfDNA and cfRNA) (Diehl et al., 2008b; Comino-Mendez and Turner, 2017; Urabe et al., 2019; Quirico and Orso, 2020; Lo et al., 2021; Wada et al., 2021; Joosse and Pantel, 2022) proteins (Martinez-Garcia et al., 2017; Signore et al., 2021), metabolites (Lee et al., 2020; Maslov et al., 2022) and lipids (Saito et al., 2021; Wolrab et al., 2022). Over the past twoades, thanks to the great advancement in sequencing technologies, a more genomic approach has been used in clinical oncology. This brought an improvement in personalized medicine in terms of prevention and treatment, providing a wide range of information on the mutational and molecular structure of many tumors (Jamal-Hanjani et al., 2016; Scherer et al., 2016; Thompson et al., 2016). The analysis of each component provides useful information on cancer diagnosis, prognosis and treatment (Lone et al., 2022). In the clinical setting, the detection of CTC above the cut-off value has a negative prognostic value in metastatic patients (Cristofanilli et al., 2004; Cohen et al., 2008) as well as the identification of a specific mutation on the ctDNA can direct therapeutic treatment or indicate drug resistance (Garlan et al., 2017). However, the exclusive use of a single omic approach does not have the power to establish all causal relationships between molecular alterations and phenotypic manifestations.

To better understand the mechanisms underlying different phenotypes during malignant transformation, investigating different omics, e.g., transcriptomics, epigenomics, proteomics and metabolomics, became necessary. Although LB represents a promising tool to monitor the dynamic evolution of cancer in a non-invasive way, its integration into clinical practice is hampered by the lack of reproducibility due to the absence of standardization across workflows (Salvianti et al., 2020). The main goal is to develop unique procedures for detection and analysis of liquid components that are reproducible and have a high degree of sensitivity and specificity.

In this review we are going to explore how the main single omics are interrogated in LB, discussing multiple types of cancers and clinical applications. At the same time, we will explain how multidimensional analysis of different liquid components and integration of omics will lead to new biomarkers discovery for cancer management, and identification of therapeutic targets linked to cancer-specific molecular pathways.

## Liquid biopsy elements

### Circulating tumor cells

Described for the first time in 1869 by Ashworth, CTCs are tumor cells that detach from the main tumor masses and travel through the circulatory systems (Pantel and Speicher, 2016). It took researchers more than a century to understand that they represent the seed for metastasis (Nguyen et al., 2009; Eslami-S et al., 2022). Epithelial to mesenchymal transition (EMT) properties and stemness features allow their dissemination in distant organs (Ye and Weinberg, 2015; Fares et al., 2020). CTCs preserve tumor heterogeneity and mimic cancer properties: for this reason, they can be used as clinical biomarkers for disease screening, dynamic monitoring and prognosis prediction (Pantel et al., 2013; Jamal-Hanjani et al., 2015; Keller and Pantel, 2019).

CTCs are related to tumor stage (Ankeny et al., 2016; Cristofanilli et al., 2019) but their clinical utility in cancer screening and/or early detection is still under debate. CTCs prognostic value has been amply proven, and their enumeration in some metastatic tumors became an independent prognostic factor (Cristofanilli et al., 2004; Cohen et al., 2008). In addition, both the presence and the size of CTC clusters can be associated with a worse clinical outcome than single CTCs (Chang et al., 2016; Paoletti et al., 2019; Lim et al., 2021).

CTCs molecular phenotype has also a strong prognostic value: for instance patients with CTC expressing mesenchymal markers (Armstrong et al., 2011), stem markers (Lecharpentier et al., 2011) or antigens such as PD-L1 (Kong et al., 2021), HER2 (Müller et al., 2021), CD47 (Agelaki et al., 2019) undergo reduced Progression Free Survival (PFS) and Overall Survival (OS). Also CD44 and CD77 have been lately proposed as prognostic markers of brain metastases (Loreth et al., 2021).

To predict and monitor therapeutic responses, CTCs can be used in combination with serum biomarkers and imaging, as demonstrated by several clinical studies. For example, patients with Castration-Resistant Prostate Cancer (CRPC) expressing androgen receptor 7 splice variant (AR-V7) protein on CTCs had a better survival rate after taxane chemotherapy (Graf et al., 2020). Expression of PDL1 on CTC seems to be a promising predictive biomarker of treatment response when using immunotherapy in Non-Small Cell Lung Cancer (NSCLC) (Kloten et al., 2019). CTC genomic aberrations and surface protein alterations can be used for monitoring tumor resistance to therapeutic regimen in breast (Gasch et al., 2016; Jordan et al., 2016), lung (Maheswaran et al., 2008; Sundaresan et al., 2016; Chang et al., 2021) and prostate cancer (Darshan et al., 2011; de Bono et al., 2021).

CTCs are difficult to detect due to their low amount and the paucity of standardized detection strategies (Table 1). To sort out this issue, usage of CTC-derived xenograft (CDX) models and CTC-derived *ex vivo* cultures have been suggested: these models represent an opportunity to identify drug susceptibility changes in patients as their tumors evolve (Hodgkinson et al., 2014; Lee et al., 2015; Suvilesh et al., 2022), as well as to understand the role of CTCs in metastatic process and metastases organotropism (Klotz et al., 2020; Bowley and Marchetti, 2022).

**TABLE 1:** Main technologies for detection of circulating tumor cells.

Principle	Technology	Enrichment	Advantage	Disadvantages	Ref
Nucleic acid-based	Targeted analysis (qPCR/NGS)	None	• High sensitivity	• No morphological analysis	Diehl et al. (2006)
			• Small volume of sample required	• Technical issues for RNA degradation	García-Foncillas et al. (2018)
					Hashimshony et al. (2016)
Protein-based	Immunocytochemistry/Immunofluorescent	• Membrane filtration	• Quantification and morphological analysis	• Subjective picture evaluation	Liu et al. (2019b)
		• Size-based enrichment	• Multi-markers evaluation	• Time-consuming	Ruano et al. (2022)
		• Immunomagnetic enrichment			Brady et al. (2020)
					Peeters et al. (2014)
	Flow Cytometry	None	• Rapid	• Low sensitivity	Van Paemel et al. (2021)
			• Multiparametric analysis	• Large sample volume requirement	Gordevičius et al. (2018)
			• High specificity	• No morphological analysis	Vrba et al. (2020)
			• Potential to sort CTCs for subsequently analysis		
	Cell-Search System	None	• Automated	• EPCAM-dependent	
			• Quantitative	• Partial subjective picture evaluation	
			• High sensitivity	• No downstream analysis	Begum et al. (2011)
			• Highly reproducible		Cohen et al. (2008)
			• Moderate sample volume		Hulbert et al. (2017)
			• FDA approval		
Microfluidic-based	DEPArray system	• Immunomagnetic enrichment	• Automated	• Subjective picture evaluation	
		• Size –based enrichment	• High sensitivity	• Time-consuming	Xu et al. (2019)
		• Cell-Search system	• Morphological analysis	• High cell loss during sample preparation	Huang et al. (2021)
			• Single cell isolation		Garvin et al. (2015)
			• Downstream genomic and transcriptomic analyses		
	CTC Chip	None	• High sensitivity and specificity	• EPCAM-dependent	
			• Quantitative	• Subjective evaluation	Chang et al. (2021)
			• Minimal manipulation of sample		Chen et al. (2021)
			• CTCs recover for further analysis		Chiu et al. (2019)
Functional-based	EPISPOT	• Size-based enrichment	• High specificity	• Detect on CTCs viable	
		• Immunomagnetic enrichment	• Quantitative	• Marker dependent	Guo et al. (2023)
			• Multiparameter analysis	• Time consuming	Månsson et al. (2021)

### Circulating tumor DNA

Since its observation in human plasma in the late 1940s (Mandel and Metais, 1948), cfDNA has received enormous attention as a noninvasive disease biomarker. Unlike cfDNA, circulating tumor DNA (ctDNA) is present in very low concentrations, ranging from ≥ 5%–10% in late stage, to ≤ 0.01%–0.1% in early stage cancers (Bettegowda et al., 2014) and has a greater degree of fragmentation. Several studies demonstrate how the length of the fragments can be linked to the mechanism of release into the circulation. Indeed, while short fragments (<200 bp) are released during apoptosis, large fragments (>200 bp) originate during necrosis (Jiang and Lo, 2016) or from viable cancer cells of primary tumors and/or metastases.

By using ctDNA we can detect specific cancer-related alterations, including point mutations, copy number variations (CNVs), and methylation changes that provide valuable insight into disease status. There is an open debate in the scientific community about the use of ctDNA for early cancer detection (Fiala and Diamandis, 2018). Grail is a company developing an Artificial Intelligence (AI)-aided early cancer detection test based on cfDNA analysis. In colon cancer (CC), the absence of ctDNA after surgery has been shown to be associated with a better prognosis and a low chance of recurrence (Schraa et al., 2022). Tracking alterations in ctDNA also allow us to detect minimal residual disease and predict recurrence several months in advance, as seen in patients with colon (Schraa et al., 2022), breast (Coombes et al., 2019) lung (Otsubo et al., 2019), ovarian (Lin et al., 2019) and prostate (Wyatt et al., 2016) cancer.

Over the last decade, the Food and Drug Administration (FDA) has approved a series of LB tests based on ctDNA. The Cobas EGFR Mutation Test v2 (Roche, Basel, Switzerland) is a diagnostic test for EGFR tyrosine kinase inhibitor therapies in NSCLC, while Guardant360 CDx (Guardant Health, Lansdale, PA, USA) and Liquid CDx (Foundation Medicine, Cambridge, MA, USA), analyzing the complete tumor genomic profile, help clinicians to understand responsiveness to checkpoint inhibitors and targeted therapy. In the near future, it will be necessary to validate non-invasive methods to detect limited amounts of ctDNA to guide personalized therapeuticisions.

### Exosome

Exosome-based oncology research has recently achieved impressive results by offering potential tools for the clinical management of the disease. Initially considered a cellular waste disposal system, they are important players in intercellular communication (Maia et al., 2018).

They are small extracellular vesicles released from every type of cell which circulate stably in most biological fluids such as blood, urine, milk, saliva. They carry nucleic acids, proteins and lipids and can reach very distant cells influencing their biological functions (Zhang et al., 2019).

In cancer exosomes are involved in remodeling of the tumor microenvironment (Huang et al., 2022a), formation of pre-metastatic niches (Costa-Silva et al., 2015) and immune-escape (Chen et al., 2018).

In liquid biopsy, for their unique features, exosomes have complementary and potentially broader applications than CTCs and ctDNA. Melo et al. (2015) found high levels of GPC1+-circulating exosomes in Pancreatic Ductal Adenocarcinoma (PDAC) patients compared to healthy controls, suggesting a strong correlation between GPC-1 exosome and cancer. Other recent studies suggested the prognostic role of circulating exosomal microRNA (miRNA). In CRPC patients, high levels of miR-1290 and -375 were significantly associated with poor OS in the follow-up cohort (Huang et al., 2015a). Besides early diagnosis and prognosis, exosomes also play a potential role in treatment response assessment. In metastatic melanoma patients, the increase in circulating exosomal PD-L1 during the early stages of pembrolizumab treatment might reflect the presence of successful antitumor immunity elicited by anti-PD-1 therapy (Chen et al., 2018). Del Re and collaborators (Del Re et al., 2017) proved that the presence of AR-V7 in exosomal RNA was associated with shorter OS and resistance to hormone therapy in CRPC patients. Despite the tangible therapeutic potential of exosomes, the lack of standardized protocols and universally accepted markers for quality control limits their use in the preclinical setting.

During exosome analysis, the real challenge to overcome is discriminating tumor exosomes from non-tumor extracellular vesicles still. A possible solution could be using highly specific analytical techniques such as mass spectrometry (MS), Flow Cytometry (FC) and High Throughput Sequencing (HTS). Big data analysis would then be capable of distinguishing tumor exosomes from the physiological material (Nguyen et al., 2019).

### microRNA

To date, the majority of circulating nucleic acid studies in oncology concern ctDNA. However the spotlight has turned on cfRNA which has promised to improve cancer diagnosis and treatment (Anfossi et al., 2018). Like exosomes, cfRNA can derive from cancerous cells, but also from non-tumor components such as stroma and the immune system. The analysis of cfRNA dynamically reflects the changes that occur in the tumor microenvironment, revealing important intercellular signaling that can be exploited in clinical practice (Anfossi et al., 2018). In the field of cfRNA biomarkers, most studies have focused on miRNAs for their greater stability in biological fluids (Glinge et al., 2017). miRNAs are a family of small non-coding RNAs that regulate a wide array of biological processes including carcinogenesis (Lu et al., 2005). They circulate in the blood carried by exosomes, apoptotic bodies or protein-miRNA complexes, functioning as either oncogenes or tumor suppressors depending on the conditions (Bartel, 2004).

In the last years, several studies demonstrated that miRNA expression is dysregulated in cancer, and its signatures could be used for diagnosis, prognosis and therapeutic management of cancer (Bartel, 2004; Reda El Sayed et al., 2021). Recently, more miRNAs have been investigated such as early diagnostic and prognosis markers for lung cancer often associated with biomarkers such as CEA and Cyfra 21-1 (Liu et al., 2021a). miRNAs may also be used to evaluate therapeutic outcomes. An example the chemotherapy increases the serologic concentration of miRNAs in colon cancer patients (Hansen et al., 2015). However, what emerges from all the studies is a high variability as more miRNAs can be associated with the same tumor with a different sensitivity and specificity. This can be partly explained by the differences in the samples tested, in terms of ethnicity of the courts, the use of endogenous controls, the biofluid analyzed, but above all on the detection method used. Consequently, standardization of procedures is needed to take full advantage of miRNAs as cancer biomarkers (Cabús et al., 2022).

### Tumor-educated platelets

The interaction between tumors and platelets was first observed in 1868 when Trousseau noted that spontaneous coagulation was common in cancer patients. A decade later, Billroth described that these clots contained cancer cells and for this reason could be agents of metastasis (Billroth, 1871). Today we know that platelets, besides carrying out protein synthesis in the absence of a nucleus, continuously exchange nucleic acids and circulating proteins with the tumor and its microenvironment (In ’t Veld and Wurdinger, 2019). TEPs are the result of this exchange which can be achieved by the direct recruitment of tumor components or indirectly by post-transcriptional splicing (Roweth and Battinelli, 2021). Thus far, the preliminary data strongly suggest that TEP-derived mRNA onco-signatures may be harnessed for cancer diagnostics, with many potential applications (Best et al., 2018). Best et al. compared TEPs derived from healthy donors and both treated and untreated patients with early, localized, or advanced metastatic cancer. They reported that molecular interrogation of blood platelet mRNA can offer valuable diagnostics information for all cancer patients analyzed—spanning six different tumor types. Platelets may be employable as an all-in-one biosource to broadly search molecular traces of cancer and provide a strong indication on tumor type and molecular subclass (Best et al., 2015). Their involvement in metastasis generation has been recently studied: TEPs seem to contribute to the survival of CTCs by protecting them from immune attacks and shear stress, promoting CTC intravenous extravasation (Liu et al., 2021b; Pereira-Veiga et al., 2022).

All in all, several liquid biopsy markers have been characterized for their clinical relevance in informing about disease biology, treatment response and progression. Each of those makers has specific strengths and limitations, and it is apparent that their combined use could be instrumental in obtaining a comprehensive description of tumor features.

For example, CTCs are informative of the tumor phenotype and genotype, representing a source of information about tumor heterogeneity and presence of molecular targets for therapy.

CTC analysis anyway poses some limitations mostly related to their difficulty to be isolated due to their limiting number in the bloodstream. On the contrary, ctDNA is informative of tumor genotype, and also of its epigenetic profile (e.g., fragmentation profile, methylation status) and is therefore useful to assess mutational profile, clonality, and tissue-of-origin. Anyway, some limits in sensitivity and specificity due to presence of other cfDNA sources and limited ctDNA quantities are still present.

Relatively new markers such as exosomes and platelets are a great promise for liquid biopsy as they hold information about relevant tumor biomolecules (nucleic acids, proteins) instrumental to retrieve information about tumor biology and microenvironment. Their combined analysis with more established markers such as CTC and ctDNA can help in obtaining a more comprehensive picture of tumor of origin, with clinical impact in diagnosis and monitoring.

## Genomics

### Genomic analysis

Tumor genetic profile is assessed in the clinical routine by molecular characterization of biopsy specimens. Due to the invasive nature of the procedure, limitations in longitudinal monitoring and intrinsic sampling biases, LB represents a valuable alternative tool to investigate the genomic landscape of tumors during time (Crowley et al., 2013). The two main sources of DNA to be evaluated in LB are ctDNA and CTCs. They both give a snapshot of the tumor mutational profile at a defined moment of the disease history. Specifically, CTCs and ctDNA can inform about point mutations, CNVs and genomic rearrangement (Tan et al., 2016). The degree of fragmentation of ctDNA and the scarce amount of genomic material contained in CTCs are factors influencing the method of choice for the downstream genomic analysis, in order to avoid bias-related amplification and to retain biological information.

Available techniques to assess the genomic profile of ctDNA and CTCs can be divided into targeted and whole-genome, targeted approaches analyze the mutational profile of a subset of genes that have been linked to the pathogenesis of disease and include clinically actionable genes of interest. Such analysis can be performed using droplet digital PCR (ddPCR) (Hindson et al., 2013) or Next Generation Sequencing (NGS)-based approaches based on amplicon sequencing such as in Tagged-Amplicon Deep Sequencing (TAm-Seq) (Forshew et al., 2012), Next Generation-Targeted Amplicon Sequencing (NG-TAS) (Gao et al., 2019) or hybridization-based capturing as in Cancer Personalized Profiling by Deep Sequencing (CAPP-Seq) (Table 2). Several commercially available panels are focused on specific gene sets and can be either specific for a tumor type or pan-cancer, covering up to hundreds of regions. A more comprehensive targeted analysis can be focused on the entire set of expressed genes by Whole-Exome Sequencing (WES).

**TABLE 2:** Overview of techniques for point mutations and CNVs detection in liquid biopsy.

Assay	Biomarker	Principle	Technology	Mutation	Advantages	Limitations	References
ddPCR; BEAMing	cfDNA CTCs; cfDNA	water-oil emulsion droplet based amplification; beads emulsion amplification	Droplet-based Digital PCR	point mutations CNVs	low input required absolute quantification	limited number of assays	Tucci et al. (2020); Diehl et al. (2006); García-Foncillas et al. (2018)
TAm-Seq	cfDNA	short, overlapping amplicon based amplification	NGS-based targeted deep sequencing	low frequency point mutations	low input required highly fragmented input	less comprehensive compared to other NGS-based methods	Forshew et al. (2012); Nikanjam et al. (2022)
CAPP-Seq	cfDNA	biotinylated oligonucleotides-based target amplification	NGS-based targeted deep sequencing	point mutations CNAs	low input required multiple assays high sensitivity	inefficient fusions detection	Nikanjam et al. (2022); Newman et al. (2014)
DOP-PCR	CTCs	degenerate oligonucleotide-primed based amplification	low-pass whole genome amplification (LP-WGA)	CNVs	very low input optimal for pre-amplification	low uniformity of coverage higher amplification-related errors	Telenius et al. (1992)
MDA	CTCs	multiple displacement amplification	low-pass whole genome amplification (LP-WGA)	CNVs	low input required high efficiency and fidelity of φ29DNA polymerase	allelic dropout, preferential amplification	Dean et al. (2001)
MALBAC	CTCs	multiple annealing and looping based amplification	low-pass whole genome amplification (LP-WGA)	CNVs	low false positive rate low chimera rate	lower polymerase fidelity	Zong et al. (2012)

DDPCR is particularly indicated for rare mutation detection or to perform CNVs analysis (Li et al., 2019b). It is highly sensitive for detecting and quantifying low levels of ctDNA (Hindson et al., 2013) even when starting from a very limited amount of input DNA or searching for residual disease. A derivation of ddPRC, Beads, Emulsion, Amplification, Magnetics **(**BEAMing) (Diehl et al., 2008b) exploits emulsion PCR combined with flow cytometry to identify and quantify specific somatic mutations present in cfDNA. Basically, target DNA regions are amplified by specific primers, then encapsulated in droplets where each single fragment gets amplified by primers covalently attached to a magnetic bead. These DNA-coated beads are purified and labeled with fluorescent probes (one for mutant, the other one for wild-type) and finally analyzed by flow cytometry (Vessies et al., 2020).

As for the NGS-based approaches, CAPP-Seq was first time reported to study ctDNA in NSCLC by Newman et al., proving high sensitivity and subsequently applied to other cancer types (Newman et al., 2014; Nikanjam et al., 2022). This approach exploits bioinformatically designed biotinylated oligonucleotides called “selectors” to specifically target exons of recurrent mutated driver genes of specific tumors. The method shows the advantage of analyzing many regions at the same time, compared to ddPCR. CAPP-seq can not only be applied to study point mutations, but also CNVs while presenting limitations in fusion detections (Newman et al., 2014; Nikanjam et al., 2022).

TAm-Seq allows the amplification of specific regions of interest through the generation of short and overlapping fragments of about 150–200 bases, then indexed singularly and deep sequenced. This technique is particularly indicated for the detection of cancer mutations with an allele frequency up to 2%. Due to the short length of amplicons generated, the method is suitable for the analysis of highly fragmented ctDNA (Forshew et al., 2012; Li et al., 2019b; Nikanjam et al., 2022). The latest version of TAm-seq, Enhanced TAm-Seq (Gale et al., 2018; Xiao et al., 2023) leverages on multiplexed PCR and reduced the detection limit to 0.02% Allele Frequency (AF) with high per-base specificity (99.9%) using as low as 6.6 ng input cfDNA. Whole genome sequencing (WGS) approaches provide a more comprehensive view of genome aberration than targeted or WES since it includes all intronic sequences, including non-coding variations. This increased resolution over the genome requires a great amount of sequencing, making WGS the most expensive of all the described approaches. The advantage of targeted approaches is indeed the great depth of information for a fraction of the cost, which is relevant in clinical diagnosis setting.

For CTCs analysis, due to the low input single cell DNA amount, whole genome amplification (WGA) is necessary to achieve enough genomic material suitable for library preparation and sequencing. The main WGA techniques utilized are: Degenerate Oligonucleotide-Primed (DOP-PCR) (Telenius et al., 1992), Multiple Displacement Amplification (MDA) (Dean et al., 2001), Multiple Annealing and Looping Based Amplification Cycles (MALBAC) (Zong et al., 2012) (Table 2). Those techniques differ in the approach used for amplification and therefore have specific advantages and limitations. The DOP-PCR method consists of a two-step exponential amplification with random primers; this approach suffers from low uniformity of genome coverage and amplification. This limit is overcome in MDA and MALBAC (Huang et al., 2015b). MDA exploits high-fidelity polymerase Φ29 to perform displacement amplification with hexamer random primers (Spits et al., 2006) and performs better in terms of false positive rate and chimera rate compared to MALBAC and DOP-PCR, thus being the preferable method for structural variation detection (Huang et al., 2015b). On the contrary, MALBAC uses quasi-random primers to perform quasi-linear amplification, thus avoiding amplification related bias, reducing allele dropout and increasing coverage uniformity (Huang et al., 2015b; Zhou et al., 2020). For this reason, MALBAC is the better method to perform CNV analysis (Huang et al., 2015b). After WGA, libraries are often sequenced at low coverage (low pass; e.g., from 0,1X coverage) to profile CNVs at a fraction of the price for high coverage. To perform mutational analysis and complex structural variant analysis a higher coverage is needed.

Depending on the type of genomic alteration to be analyzed, e.g., CNVs, Insertion–deletion (indel) or single nucleotide variants (SNV), different metrics are required. For example, CNVs analysis requires high coverage uniformity, while for indels and SNV detection, low rate of amplification errors and allele dropout are particularly critical (Lu et al., 2020).

### Clinical applications

Until now, most clinically validated LB tests are based on the assessment of actionable genomic alterations with the aim of supporting decisions about targeted cancer therapy and monitoring treatment response (Ou et al., 2018). However, with the advancement of sequencing platforms that enable more sensitive detection of different classes of mutations, genomic approaches on circulating biomarkers are expanding the range of potential applications in cancer management.

Currently, the most promising application of LB is cancer screening and early detection. Compared to metastatic setting, cancer in early stage releases a low amount of circulating tumor material and thus their detection requires highly sensitive and specific techniques. Moreover, recent reports of somatic mutations accumulating both in solid tissues and in the hematopoietic system as a function of age are reported (Genovese et al., 2014; Alexandrov et al., 2015). In this regard, the company GRAIL, Inc., (https://grail.com) is developing a ctDNA-based multi-cancer screening test using advanced NGS approaches and Machine Learning (ML) (Aravanis et al., 2017). Its first multi-center clinical study, the Circulating Cell-free Genome Atlas (CCGA) will analyze samples from 10,000 participants between cancer and in healthy donors with the aim of identifying a specific genomic signature that distinguishes healthy from sick at an earlier stage of disease. From earliest data, SNV and somatic copy number alteration (SCNA) showed statistically worse sensitivity than WG methylation (Jamshidi et al., 2022). On the contrary, in another study, Manier et al. performed Low-pass WGS (LP-WGS) to study SCNAs in CTCs and ctDNA to uncover the genomic profile of multiple myeloma patients in early stages of disease. Combining the analysis of both analytes, they obtained a higher fraction of patients providing different yet complementary information regarding clonal heterogeneity (Manier et al., 2018).

Different studies exploit genomic approaches for the prognostic validation of circulating biomarkers. A large meta-study suggests that the presence of ctDNA KRAS mutations was associated with shorter OS in NSCLC (Zhang et al., 2019\). In another study, a high number of CTCs (≥20 CTC/10 mL of blood) with mesenchymal phenotype, identified with V600E mutation in BRAF, were correlated with a poor prognosis of the melanoma patients (Tucci et al., 2020). To date, the real strength of LB-based patient management is the ability to track tumor evolution for predicting and monitoring treatment response and resistance mechanisms. Recently, it has been demonstrated that the evaluation of the genomic profile of advanced BC in cfDNA can identify subclonal resistance mutations not appreciable on by single site metastatic tumor biopsies. The cfDNA analyzed with a clinical panel of 74 tumor-associated genes revealed diverse subclonal resistance mutations in specific breast subtype such as HER2 mutations in HER2 + disease, PIK3CA mutations in HR + disease or mutual exclusivity of ESR1 mutations and MAPK pathway alterations in HR + HER2 − BC subtype (Turner et al., 2020; Kingston et al., 2021). With the advent of immunotherapy, many studies have attempted to establish a reliable predictor of response to immune checkpoint blockade. In addition to PD-L1, Tumor mutational burden (TMB) of tumor tissue has been shown to correlate with response to immune checkpoint therapy (Goodman et al., 2017; Klempner et al., 2020; Li et al., 2021). However, some patients lack a high-quality tissue biopsy suitable for biomarker analyses.

To address these challenges, Foundation Medicine has developed a new test based on circulating TMB which has already demonstrated clinical validity. Data published in Nature Medicine demonstrated that the blood-TMB (bTMB) test could predict response to atezolizumab in patients with previously treated NSCLC. Furthermore, the results show that bTMB may be an independent predictor of response compared to PD-L1 expression, as assessed by immunohistochemistry in patients who also had a tissue biopsy available (Gandara et al., 2018). In other work, Georgiadis et al. developed an approach for detection of Microsatellite Instability (MSI) and TMB in the cfDNA of late stage-cancer patients treated with PD-1 blockade. Patients with MSI and TMB-Hight tumors had improved PFS and OS (Georgiadis et al., 2019).

The use of genomic approach on ctDNA to identify resistance alterations to treatment still represents the only validated application in clinical practice. The ctDNA analysis of T790M-mutant NSCLC patients, resistant to first line EGFR Tyrosine Kinase Inhibitor (TKI) therapy, revealed different patterns of genetic alterations in patients with innate versus acquired resistance to Osimertinib (Kato et al., 2021). BRCA reversion mutations detected in cfDNA of platinum-resistant high-grade ovarian carcinoma (OC) patients were associated with decreased clinical benefit from enzyme poly ADP ribose polymerase (PARP) inhibitor rucaparib. Besides, ctDNA analysis identified multiple BRCA reversion mutations, indicating the ability to capture multiclonal heterogeneity of this tumor (Georgiadis et al., 2019). Several key studies have further highlighted the transformative impact of LB for Minimal Residual Disease (MRD) detection, illustrating the future clinical potential and real-world impact (Tie et al., 2016; Schøler et al., 2017; Coombes et al., 2019). A recent paper by Parikh et al. evaluated the feasibility of MRD detection with a plasma ctDNA assay in CC patients with stage I–IV undergoing curative-intent surgery (Parikh et al., 2021). Of 70 patients with eligible plasma for testing, 17 of 70 patients (24%) were ctDNA positive after completion of therapy and 15 of these patients recurred while standard serum carcinoembryonic antigen levels were not predictive of any recurrence.

## Transcriptomics

### Transcriptomic analysis

Transcriptome analysis (both coding and non-coding RNAs) in LB represent a valuable source of biomarker for precocious identification, stratification and prediction of tumor outcomes (Cuzick et al., 2011). In particular, miRNA, other than their biological role as post-transcriptional regulators, have been found to be dysregulated in several cancer types (Lu et al., 2005; Fernandez-Mercado et al., 2015). miRNAs are protectively released by tumor mass in association with RNA-binding proteins or included in microvesicles and are involved in oncogenesis and tumor progression through their ability to regulate expression of specific genes (Pinzani et al., 2021). Their increased stability and resistance to degradation, compared to mRNA, allow easier analysis of miRNA in body fluids (Mitchell et al., 2008). The analysis of mRNA from plasma is particularly challenging due to its limited stability and quantity. Extracellular mRNA is indeed highly fragmented by ribonucleases. This is reflected in few transcriptome-wide studies (Li et al., 2022) and more targeted approaches intended to capture selected miRNA or mRNAs (Qu et al., 2017). Reverse transcription associated with PCR (e.g.,.ddPCR or qPCR) is actually the method of choice to interrogate circulating tumor RNAs (Pinzani et al., 2021; ruyenaere et al., 2021). For instance, the application is suitable for the analysis of specific cancer mutated genes such as PD-L1 whose expression is evaluated in many tumor types (Ishiba et al., 2018; Pinzani et al., 2021).

A more comprehensive view of circulating and CTC RNAs is provided by NGS-based approaches.

Indeed, it is possible to profile mRNAs using methods that enrich poly(A) RNAs, or selectively reverse transcribe them. In case of non-coding RNAs, sequencing libraries can be obtained from total RNA as well as after enrichment for short transcripts.

To profile the reduced quantity of RNA present in CTCs, several single cell analysis methods have been developed, e.g., Smart-Seq (Picelli et al., 2013), CEL-seq (Hashimshony et al., 2012) and STRT-Seq (Islam et al., 2011) (Table 3). The Smart-seq technology is considered the gold standard for single cell RNA-seq thanks to its higher sensitivity, accuracy and lower costs. In comparison with CEL-seq technology, Smart-seq shows higher sensitivity for the detection of higher number of genes per cell and lower dropout rate (Ziegenhain et al., 2017).

**TABLE 3:** Overview of techniques to study gene expression changes in liquid biopsy.

Assay	Biomarker	Principle	Advantages	Limitations	References
Smart-Seq	CTCs	polyA enrichment, no UMI, full length by template switch	high sensitivity low allele dropout	applicable to polyadenylated RNA	Picelli et al. (2013)
CEL-seq, CEL-seq2	CTCs	polyA enrichment, UMI, 3′ counting	low input, sample pooling and barcoding to process single cell in a time	3′ related bias, preferentially amplified transcripts	Hashimshony et al. (2012)
					Hashimshony et al. (2016)
STRT-Seq	CTCs	polyA enrichment,UMI, 5′ tag counting	sample pooling and barcoding to process single cell in a time	amplification related errors	Islam et al. (2011)
small RNA-seq	CTC, cfRNA	two adaptor-ligation; polyadenylation and template switching	none	high background	Roman-Canal et al. (2019)

### Clinical applications

Over time, it became clear that direct analysis of circulating RNAs could help better understand the evolutionary dynamics of cancer, by providing useful insights for developing personalized approaches for tumor diagnosis and therapy (Supplitt et al., 2021). Several studies demonstrate that miRNAs alone or in combination with other biomarkers improve the diagnostic and prognostic power of different tumors (Chan et al., 2013; Hou et al., 2016; Roman-Canal et al., 2019; Zhang et al., 2022). An interesting study performed cell-free RNA and exosome-RNA analysis on 44 early-stage PDAC patients, identifying 13 upregulated miRNA in PDAC patients compared to healthy controls. A combinatorial analysis of cell-free and exosomal miRNA identifies a particular signature able to detect patients at an early stage of the disease, thus evidencing the diagnostic power of the test (Nakamura et al., 2022).

Furthermore, Sabato and colleagues recently identified specific upregulated and downregulated circulating plasma EV linked to microRNA expression in metastatic melanoma patients compared to healthy donors. They bioinformatically identified 4 pEV-microRNAs able to distinguish metastatic patients from healthy controls with a high diagnostic potential (Sabato et al., 2022). In addition to early diagnosis and prognosis, miRNAs have also been tested for predictive ability to patients’ therapeutic response and cancer resistance in a broad category of tumor types (Hon et al., 2018; Niwa et al., 2019; Tian et al., 2019). In a study of 43 BC patients, the authors identified a clear association of four pEV miRNA with a pathological response to neoadjuvant therapy while no correlation between the miRNA transcriptional profile performed on plasma compared to tissue biopsy one (Baldasici et al., 2022). In a recent study, exosomal miRNAs were shown to participate in osimertinib resistance through abnormal activation of the RAS-MAPK and PI3K pathways. Particularly the expression of miR-184 and miR-3913-5p in the peripheral blood of NSCLC patients could be used as biomarkers to indicate osimertinib resistance (Giallombardo et al., 2016). RNA-seq of single prostate CTCs from patients resistant to Androgen Receptor (AR) inhibitor showed activation of noncanonical Wnt signaling and low glucocorticoid receptor expression compared with untreated cases (Miyamoto et al., 2015). Furthermore, the transcriptomic profile of CTCs was analyzed to identify a specific subpopulation involved in the metastatic spread and organotropism of different cancer types (Giuliano et al., 2014; Castro-Giner and Aceto, 2020; Schuster et al., 2021; Yu et al., 2021).

It has been shown that CTC-derived cell cultures and xenograft models could more accurately define cell clones with an initial metastatic long-term potential (Vishnoi et al., 2019; Brungs et al., 2020; Felici et al., 2022). Analyzing CTCs isolated from breast patients, Boral et al. identified a specific signature associated with brain metastases (Boral et al., 2017). An interesting work studied the spatiotemporal transcriptional dynamics of CTCs during hematogenous dissemination in patients affected by hepatocellular carcinoma. The authors identified specific CTC phenotypes in different vascular compartments involved in liver tumor dissemination. They observed an overexpression of chemokine CCL5 involved in CTC immune escape and metastatic seeding mediated by recruitment of regulatory T cells (Tregs). These findings open the way to directly block CTC dissemination through the inhibition of CCL5, with the possibility to extend the target to other driver genes involved in immune evasion (Sun et al., 2021).

Elsewhere, CTCs isolated from metastatic gastric cancer (GC) patients presented upregulation of genes involved in platelet adhesion. The transcriptomic profile of CTCs demonstrated the important contribution of platelets in EMT progression and acquisition of chemoresistance (Negishi et al., 2022). Taken together, these results represent an important step in developing effective strategies against CTCs to prevent cancer dissemination and immune evasion.

## Epigenomics

### Epigenomic analysis

Epigenetic alterations include DNA methylation, histone modification and chromatin accessibility to transcription factors. By determining gene expression patterns, those modifications shape cellular phenotype. Alterations resulting in an overly permissive or overly restrictive epigenetic regulation can lead to the generation of malignant subclones responsible for tumor progression and therapeutic resistance (Ponnusamy et al., 2019; Fujimura et al., 2020; Chen and Yan, 2021). For this reason, epigenetic alterations have been proposed as potential diagnostic, prognostic and predictive biomarkers in cancer management (Heyn and Esteller, 2012). Detection of epigenetic modifications of cfDNA and CTC using LB can reveal the epigenetic aberration of cancer.

DNA methylation is the most studied epigenetic modification. Silencing of tumor suppressor genes by extensive promoter hypermethylation has been observed in several cancers (Greger et al., 1989; Herman and Baylin, 2003; Chen et al., 2014). DNA methylation analysis can be performed using three experimental approaches: bisulfite conversion, digestion with methylation-sensitive restriction enzymes and affinity enrichment (Table 4) (Galardi et al., 2020).

**TABLE 4:** Overview of techniques to study epigenetic alterations in liquid biopsy.

Assay	Biomarker	Principle	Technology	Alteration	Limitations	Advantage	References
WGBS, LCM–µWGBS	ctDNA, CTC	Bisulfite conversion and sequencing Laser capture microdissection and BS-seq	Whole genome, single base resolution	DNA methylation (CpG sites)	Sequencing cost, high input required. Does not discriminate hmeC form meC	Work with limited input quantities	Hai et al. (2022)
							Gkountela et al. (2019)
							Zhao et al. (2021)
RRBS, cfRRBS	ctDNA	Bisulfite conversion, enzymatic digestion to enrich CCGG sites	CpG rich regions, single base resolution	DNA methylation	Only probes high density CpG regions. Does not discriminate hmeC form meC	Reduced cost	Meissner et al. (2005)
							Van Paemel et al. (2021)
Methylation array	ctDNA	Bisulfite conversion and hybridization on array	Bisulfite conversion, single base resolution	DNA methylation	High input required. Does not discriminate hmeC form meC	Targeted on functionally relevant sites; simplified readout	Gallardo-Gómez et al. (2018)
							Gordevičius et al. (2018)
methylation-specific PCR (MSP)	ctDNA	Bisulfite conversion, targeted amplification followed by qPCR/real-time PCR quantification or NGS	Targeted, single base resolution	DNA methylation	Probe design, input quantity. Does not discriminate hmeC form meC	Easiest approach for a limited target number	Vrba et al. (2020)
							Begum et al. (2011)
							Hulbert et al. (2017)
meDIP-seq; cfMeDIP-seq	ctDNA	Enrichment by antibody	Affinity-based enrichment, low resolution	DNA methylation	Input material, recovery of low density CpG regions	More conservative than bisulfite conversion, cheaper than WGBS. Discriminate meC from hmeC	Zavridou et al. (2020)
							Xu et al. (2019)
MBD-seq; cfMBD-seq	ctDNA	Enrichment by methyl binding protein	Affinity-based enrichment, low resolution	DNA methylation	Input material, recovery of low density CpG regions	More conservative than bisulfite conversion, cheaper than WGBS. Discriminate meC from hmeC	Huang et al. (2021)
							Huang et al. (2022b)
hMe-Seal	ctDNA	Enzymatic conversion, whole genome, works with low input	Hydroxycytosine analysis	DNA methylation	Does not probe meC	Low input required	Song et al. (2011)
							Song et al. (2017)
							Chen et al. (2021)
							Chiu et al. (2019)
EM-seq	ctDNA	TET2 and APOBEC3A to convert unmethylated cytosines	Enzymatic conversion, whole genome	DNA methylation	Sequencing cost	Comprehensive coverage of hmeC and meC	Vaisvila et al. (2021)
							Guo et al. (2023)
cfChIP-seq	ctDNA	Immunoprecipitation of cell-free nucleosomes carrying active chromatin modifications followed by sequencing. Can be combined with qPCR (targeted)	Chromatin immunoprecipitation	Chromatin: accessible/active promoters (H3K4me3 or H3K4me2), enhancers (H3K4me2 or H3K4me1) and gene body of actively transcribed genes (H3K36me3)		Probe histones modifications on limited input	Sadeh et al. (2021)
							Månsson et al. (2021)

The bisulfite conversion is the gold standard in 5-metil-cytosine (5 mC) detection and it’s based on chemical modification of unmethylated cytosines. Comparison of bisulfite-converted and reference unconverted sequences allows the identification of methylated cytosine with a single base resolution. Bisulfite conversion can be exploited for targeted analysis, when followed by Methylation-Specific PCR (MSP) or genome-wide characterization by means of Whole-Genome Bisulfite Sequencing (WGBS-seq), Reduced-Representation Bisulfite Sequencing (RRBS-seq) (Meissner et al., 2005; Gu et al., 2011) methylation arrays and Methylated CpG Tandems Amplification and Sequencing (MCTA-seq). Although WGBS offers a comprehensive profiling of GpC methylation status at a single base resolution, it is not applicable to large cohort analysis due to high costs of whole genome sequencing. RRBS instead profiles approx. 4 million of cytosines in CpG dense regions for a fraction of WGBS cost (Gu et al., 2011). Human methylation arrays such as Infinium MethylationEPIC BeadChip Kit (Illumina) instead probes 850,000 functionally relevant methylation sites at single-nucleotide resolution but requires a quite high input material (200–300 ng), thus reducing applicability to liquid biopsy studies. To overcome this limitation, pooling approaches to methylation array strategy (Gallego-Fabrega et al., 2015) have been applied to profile cfDNA of CC patients (Gallardo-Gómez et al., 2018). To apply bisulfite analysis on CTCs, single cell-RRBS (Guo et al., 2015), PCR-based and target bisulfite sequencing methods can be exploited.

As a general limitation associated with use of bisulfite is the damaging effect of chemical treatment on DNA, which is associated with material loss and requirement of moderately high starting material (Grunau et al., 2001), bisulfite-free methods for epigenomic profiling have been recently developed. For example, Methylation Restriction Enzymes method (MREs) uses methylation sensitive restriction enzymes that recognize and cut only unmethylated DNA. Quantitative analysis of methylation status after digestion can be performed using various technologies based on real-time PCR (Hashimoto et al., 2007; Pulverer et al., 2021), sequencing (Oda et al., 2009) and microarray (Hatt et al., 2015).

Other bisulfite-free assays are based on affinity enrichment either using anti-methylcytosine antibodies (meDIP-seq) (Taiwo et al., 2012) and low input, cfDNA compatible cfMeDIP-seq (Shen et al., 2019) or Methyl-Binding Domain (MBD) of methyl-CpG binding proteins to capture the methylated genomic regions (Serre et al., 2010) optimized for cfDNA (Huang et al., 2022b).

In addition to 5mC, 5-hydroxymethylcytosine (5hmC) has recently proven to be a regulatory modification associated with transcriptional activation (Song et al., 2011) and several studies identified 5hmC as putative marker in cancer (Vasanthakumar and Godley, 2015) detectable in ctDNA samples (Song et al., 2017). Standard bisulfite methods cannot discriminate between cytosine modifications, and enrichment methods often require high input material. A recently developed technology called Enzymatic Methyl-seq (EM-seq) allows the identification of both 5mC and 5hmC starting from picograms of material exploiting two sequential enzymatic reactions (Vaisvila et al., 2021). Another innovative method for studying genome-wide 5hmC is 5hmC-Seal (Song et al., 2011) which uses a selective chemical labeling on low levels of DNA with high sensitivity.

Finally, a recently developed technological approach for detecting cancer-specific methylation and cancer-associated fragmentation signatures, without disrupting bisulfite conversion, is native Nanopore sequencing (Katsman et al., 2022; Lau et al., 2022). In this assay, PCR-free libraries are read at a single molecule level by passing through nanopores generating an alteration of electric signal which is recorded by the sequencer. This alteration is specific to the DNA modification and is different between methylated and unmethylated CpGs. By abolishing PCR, the biases in molecule quantification are strongly reduced. With this approach it is possible to cover many informative cfDNA CpG sites, even with a shallow coverage, still obtaining significant discrimination on aberrant methylation state (Lau et al., 2022) and retain cell-of-origin information (Katsman et al., 2022).

Aside from DNA methylation, chromatin accessibility and histone modifications are becoming increasingly studied in parallel with the development of new technologies for their detection in the context of liquid biopsy. Open chromatin regions undergo higher fragmentation than compacted portions which are protected by nucleosomes. Those accessible regions are associated with transcription and have been associated with a typical fragmentation pattern related to the tissue-of-origin (Snyder et al., 2016; Moss et al., 2018).

As reference chromatin accessibility profiles for many cancer types are now available (Corces et al., 2018), computational identification of key disease features to be targeted in liquid biopsies is possible, allowing computational imputation of cfDNA tissue derivation (Cristiano et al., 2019; Sun et al., 2019). That information can be integrated in panel design to prioritize coverage of marker regions with highest detection probability (Taklifi et al., 2022).

It has been observed that cancer-derived cfDNAbe more variable in length than cfDNA from non-cancer cells, due to the altered chromatin accessibility and associated nucleosome positioning. This feature of cfDNA has been exploited in the combined detection of fragmentation patterns and genetic markers resulting in improved sensitivity in cfDNA analysis (Peneder et al., 2021). This approach can additionally benefit from enrichment in short (50–150 bp) plasma derived fragments in combination with LP-WGS (Mouliere et al., 2018).

Cell-free chromatin in plasma can be further analyzed to detect nucleosomes and associated Post-Translational Modifications (PTM). Immunoprecipitation of cell-free fragments followed by low-depth NGS sequencing (cfChIP-seq) has been shown to inform about cell type and program-specific expression patterns (Table 4) (Sadeh et al., 2021). In CC patients, plasma detected levels of H3K9me3 and LINE, detected by immunoprecipitation followed by sequencing, were found to be significatively associated with disease (Gezer et al., 2013). Finally, a database of nucleosome positioning *in vivo* and of cell-free DNA nucleosomes (NucPosDB) has been recently released (Shtumpf et al., 2022). It has been built from published *in vivo* nucleosome positioning datasets together with datasets of sequenced cfDNA. This type of data represents an invaluable resource for model training especially for association of cfDNA nucleosomes to the tissue and cell of origin, and to set comparison between different conditions.

### Clinical application

Alteration of epigenetic markers in liquid biopsy has shown to be clinically meaningful although it is still affected by limited availability of standardized tests (Palanca-Ballester et al., 2021).

Most promising diagnostic applications are based on cfDNA methylation analysis, especially with custom panels, which better balance sensitivity and test cost. The PanSeer assay was developed for interrogation of cancer-specific DNA methylation signatures from peripheral blood (Chen et al., 2020). This assay targets more than 10,000 CpG sites across the genome, panel is tissue-of-origin (TOO) independent and was able to detect cancer-related aberration up to 5 years before in asymptomatic patients. Similarly, a 100,000 regions classifier based on methylation status was developed by the TCGA consortium to provide for cancer detection and TOO identification (Liu et al., 2020) in >50 cancer types. This assay has been validated on a case-control cohort and showed a >99% specificity which increases with cancer stage, and a TOO detection accurate in 90% of cases. Furthermore, recent studies have shown that the cfDNA hydroxymethylation pattern is associated with cancer type and can change in a stage-dependent manner (Song et al., 2017).

Beside those pan-cancer panels, several tumor-specific methylation patterns have been identified such as in BC in which the analysis of cfDNA methylation signature improves early detection of BC compared to mammography (Zhang et al., 2021). Whereas in glioma, the mere identification of the methylation status of the MGMT promoter in CSF rather than plasma provides a promising clinical tool for early diagnosis (Wang et al., 2015). Such targeted approach benefit from improved sensitivity when integrated with other cfDNA parameters such as fragment size analysis (Shin et al., 2022) and/or cfDNA plasma concentration to differentially methylated regions analysis (Panagopoulou et al., 2019; Bagley et al., 2021). A very recent study proposed a hydroxymethylation classifier for early stages CC in cfDNA plasma which can be expanded to DNA fragment size and abundance to increase sensitivity (Walker et al., 2022). Integration of haplotype blocks methylation analysis represents an add-on to single CpG loci analysis. Those are genomic segments of strictly related CpG sites whose methylation status is informative of tumor load and tissue-of-origin mapping (Guo et al., 2017). A haplotype load metric has been correlated with tumor load and tissue-of-origin mapping in cfDNA of lung or CC patients (Guo et al., 2017) thus representing a promising analysis tool for deconvolution and disease identification.

Epigenetic markers monitoring has also proven useful for outcome prediction and longitudinal monitoring of minimal residual disease in different cancer types. Interrogation of a panel of ctDNA methylation markers in post-surgery colon cancer patients has been shown to be predictive of recurrence with good accuracy (Jin et al., 2021). Similarly, methylation levels of specific marker USP44 has been associated with disease-free survival in prostate adenocarcinoma patients (Londra et al., 2021). Interestingly, WGBS approach has been used to assess the methylation profile of single CTCs and CTC clusters in BC patients, revealing specific hypomethylation of proliferation and stemness regulators binding sites only in CTC clusters. Moreover, PFS analysis showed that those regions hypomethylated in CTC clusters are associated with poor prognosis (Gkountela et al., 2019).

Methylation markers are not only significant for prognosis, but also for predicting therapeutic response. Indeed, in patients with NSCLC, increased RARB2 methylation in cfDNA after chemotherapy and tumor resection was associated with disease recurrence at 9 months (Ponomaryova et al., 2013) as well as decreased levels of plasma SHOX2 methylation was associated with response to platinum-based chemo/radiotherapy (Schmidt et al., 2015). In metastatic CRPC, methylation changes in specific genes were predictive of response to AR treatment (Peter et al., 2020; Peter et al., 2022). In early stage NSCLC patients, Markou et al. recently showed correlation between relapse incidence and promoter methylation status of at least one of a five selected genes panel (APC, RASSFIA1, FOXA1, SLFN11, SHOX2) in CTC or plasma-cfDNA (Markou et al., 2022). In the analyzed cohort there was no correlation between CTCs and primary tumor methylation profile.

Paired analysis of another nine-gene promoter methylation panel in ctDNA and CTC of a different NSCLC cohort, analyzed before osimertinib treatment and during progression, revealed inconsistency between CTC and ctDNA profiles. However, in both cases Progression Disease (PD) was associated with increased methylation levels with respect to baseline samples. Kaplan-Meier analysis revealed correlation between earlier PD and methylation status of at least one panel gene (Ntzifa et al., 2021). As those data were obtained on small CTC bulks by real-time methylation specific PCR assays, this can suggest the relevance of performing additional single cell analysis to deconvolve potential heterogeneity.

## Proteomics

### Proteomic analysis

Proteomic analysis of LB represents a minimally invasive and repeatable way to accomplish a broad range of milestones such as: identification of targets to direct new treatments, development and validation of biomarkers to allow early detection of diseases, design of proteomic signatures as predictive models for cancer diagnosis and prognosis (Kim et al., 2016). Using the appropriate technique to investigate proteomic signatures is becoming as important as studying more than one specimen from the same patient, to achieve a deeper level of molecular complexity.

A traditional method to validate protein biomarkers is using enzyme linked immunosorbent assays (ELISA), but this approach is time consuming, expensive and depends on the availability of existent antibodies pairs for every target protein. A method which does not rely on existing validated antibodies is MS (Kim et al., 2016). To analyze complex protein mixtures ensuring high sensitivity, MS is often coupled with gas or liquid chromatography, capillary electrophoresis, or Ultra-Performance Liquid Chromatography (UPLC). Based on the identification of the ratio of mass to charge (m/z) of a molecule and/or its fragments, the initial chromatographic step increases the isobaric species’ resolution, gaining a better detection of less abundant proteins. However, the use of chromatography increases the processing times and therefore limits the number of samples to be tested daily (Ardrey, 2003). When applicable, biofluids could be injected directly in the mass spectrometer: this method, called flow-injection MS, even if characterized by reduced sensitivity, could be advantageous for initial screenings aimed at having a general picture of the total protein amount of the sample (Sarvin et al., 2020).

A complementary analysis of both plasma and urine from the same patient allows a thorough examination since urine is a filtrate product of blood (Chinello et al., 2019). As a recent work demonstrates, label-free liquid chromatography−tandem mass spectrometry (LC−MS/MS) approach, through which liquid chromatography was coupled to tandem MS to fragment selected peptides, was used to compare soluble protein signatures of urine and plasma from patients affected by renal cell carcinoma. Some differentially expressed proteins were shared between the two biofluids such as immunoglobulin, complement factors, activators of the complement cascade, modulators of the acute response, innate immune system, and platelet degranulation. However, urine and blood carried specific biofluid functional signatures (Chinello et al., 2019).

Although extremely informative, MS results are often validated using more conventional techniques such as ELISA and Western Blotting (WB), which can be still useful to identify a few target proteins in low abundance, but without giving information about the protein expression levels (Lequin, 2005; Kurien and Scofield, 2015). Nevertheless, even these techniques are evolving into more precise and accurate methods to quantify circulating proteins and interrogate proteomic signatures at a single-cell level: multiplex ELISA and single-cell (sc)-WB (scWB) can serve as a model (Velez et al., 2021).

Differently to standard ELISA, multiplex ELISA can detect and process up to 1,000 human proteins, allowing us to develop a multiplex bioassay and add more candidate proteins into a panel of interest (Song et al., 2019). However the performance of an antibody-based approach might be affected by several variables such as abundance of the protein of interest, affinity and specificity of the capture antibody (Fu et al., 2010).

For phenotyping characterization of CTCs, scWB can be used. Among the novel single-cell immunoassays, scWB can potentially allow multiplex detection of surface, intracellular and intranuclear proteins simultaneously. Into *ad hoc* microwells all steps of WB are performed, minimizing cell loss and maximizing protein concentration (Sinkala et al., 2017). The initial electrophoretic separation reduces the antibody cross-reactivity and increases assay specificity, while antigen immobilization on the detection membrane is associated with molecular size standards. Despite the enormous potential of this method, initial steps of CTCs isolation, enrichment and transfer into micro wells can be challenging and opportune instruments are required (Abdulla et al., 2022).

It has been a few years already that single-cell proteome analysis is performed using mass cytometry (MC), which comes from the fusion of two technologies, FC and MS. MC allows in-depth analysis of homogenous cell populations, providing measurement of over 40 parameters at single cell resolution (Spitzer and Nolan, 2016) from a limited sample volume. Both surface and intracellular proteins can be targeted using FC features and antibodies against selected targets. Then, after cell nebulization and ion cloud filtration using a quadrupole, droplets can enter into the mass cytometer and enriched heavy metal reporter ions are finally quantified by time-of-flight. On this basis, a single-cell measurement can be achieved, even for rare populations, enabling the analysis of heterogeneous complex cellular systems (Frei et al., 2016). However, despite the enormous potential of this technique, this kind of analysis does not allow live cell recovery, and measurement of low expressed molecular features may fail due to the paucity of available sensitive fluorophores and the background noise.

Noteworthy, a technology that could be used for rapidly investigating surface protein expression in CTCs is the DEPArray system (Menarini Silicon Biosystems, S.p.A., Italy). Already known for its ability to isolate single, viable rare cells using dielectrophoretic principles combined with an image-based selection, DEPArray technology has gradually gained clinical relevance during the lastade (Bulfoni et al., 2016; Boral et al., 2017).

### Clinical application

It has been decades that the diagnostic, prognostic and treatment monitoring value of serum and urine tumor markers have been proved. Serum PSA levels for diagnosing prostate cancer in men, cancer antigen 15-3 (CA15-3) for breast cancer and cancer antigen 19-9 (CA19-9) for pancreatic cancer are just a few examples of how medicine has applied soluble protein dosage to diagnose cancer to date (De Angelis et al., 2007; Sturgeon et al., 2009). However, due to their reduced sensitivity and lack of cancer specificity, tissue biopsy is regularly performed to confirm the definitive diagnosis, avoiding incorrect interpretations associated with benign conditions.

Plasma and urine have been the most characterized biological source of data by far, however some biomarkers are massively diluted and alternative more concentrated clinical samples have been investigated recently. Among the broad set of biological fluids considered valuable sources for biomarker discovery, nipple aspirate fluid (NAF) is gaining an emerging role for BC screening in young women at high risk. Indeed, not only mammography has low specificity in differentiating between benign and malignant growth, but also in this scenario this test lacks accuracy due to young breast density. Sadr-ul Shaheed and collaborators (Shaheed et al., 2017) investigated the protein-rich composition of NAF using MS techniques identifying 332 new biomarkers unique to NAF. Using 2D-LC/MS, NAF proteome from BC patients and healthy volunteers was analyzed to prove the potential diagnostic value of the above cited procedure. NAF proteome already has the potential to give us plenty of data about breast health, especially for its highly rich composition of biological materials (Chan et al., 2016; Do Canto et al., 2016; Shidfar et al., 2016).

Similarly, Gabriel Vales et al. have recently validated 20 proteins using quantitative multiplex ELISA array starting from vitreous samples while looking for uveal melanoma biomarkers associated with metastatic risk (Velez et al., 2021). In this study, for the first time in vitreous, this technique was used to investigate such a large set of proteins, also confirming previous gene expression analysis. Sampling and analyzing circulating tumor markers in these fluids in a minimally invasive way is essential when tumors are critically located.

If soluble proteins’ dosage is effortless because they are easily accessible, we cannot state the same for surface, intracellular and particles’ proteins. Over the past decade, EV and particles EV (EVP), which include small exosomes, large exosomes and exomeres (Zhang and Lyden, 2019) have gained increasing importance in cancer detection from LB and beyond, since they reflect the systemic effects of cancer. Recently, the prognostic and functional importance of tumor-derived exosome’s proteins has been proved in tumor progression, immune regulation and therapy guidance (Costa-Silva et al., 2015; Hoshino et al., 2015; Chen et al., 2017; Rodrigues et al., 2019).

A recent multicentric study has elegantly investigated the proteomic profile of EVPs in 426 human samples from tissues, plasma and other bodily fluids, related to adult (pancreatic, lung, breast, and colorectal carcinomas and melanoma) and pediatric cancers (neuroblastoma and osteosarcoma) (Hoshino et al., 2020). Using MS combined with ML approach, they identified pan-EVP markers, demonstrating that tumor-associated EVP proteins are reliable biomarkers for early-stage cancer detection and determination of uncertain primary tumor types, reaching 95% sensitivity and 90% specificity.

As for many exploratory studies, Hoshino’s research group interrogated publicly available protein databases to find proteome quantitative data and then make a list of tumor specific EVP proteins of interest (Omenn et al., 2005; De Angelis et al., 2007; Mathivanan and Simpson, 2009; Kalra et al., 2012; Hoshino et al., 2015; Chan et al., 2016; Do Canto et al., 2016; Shidfar et al., 2016; Chen et al., 2017; Shaheed et al., 2017; Rodrigues et al., 2019; Zhang and Lyden, 2019; Hoshino et al., 2020). After having selected conventional and newly identified markers, they employed a targeted MS-approach, using a designed time-scheduled parallel reaction monitoring (PRM) method, to quantify tissue-specific tumor-derived EVP proteins in patients. PRM is particularly suitable for quantifying tens to hundreds of targeted proteins in complex matrices with attomole-level limit of detection. Tumor-associated EVP protein profiles could serve as a LB tool to detect cancer and discriminate among heterogeneous cancer types, as also confirmed by EVP profiles of tissue biopsies (i.e., lymph nodes).

Similarly, Yunee Kim and his collaborators used targeted proteomics combined with computational biology to define proteomic signatures for prostate cancer from urines collected by men with extra prostatic and organ-confined prostate cancer, in a 74-patient cohort. Since protein signatures, rather than individual soluble proteins, allow us to accurately discriminate patient groups, they tried to distinguish pT2 stage from pT3 stage tumors, before radical prostatectomy, for potentially modifying and personalizing patient treatment (Kim et al., 2016). Selected Reaction Monitoring Mass Spectrometry (SRM-MS) allows targeted quantification of a large number of proteins in a selective and sensitive way (Kim et al., 2016). A ready-to-use prognostic signature could help in the clinical decisions-making process leading to appropriate treatments, improving survival. If to date CTCs enumeration alone has been a new method to diagnose cancer, monitoring surface and intracellular protein expression in CTCs, together with transcriptomic and genomic analyses, is going to be the next step to enhance prognostic decisions, classify patients in low and high-risk groups, and better guide treatments. scWB and MC could both satisfy this clinical need (Stelzer et al., 2021). Indeed, E. Sinkala and his collaborators, within a pilot study, used scWB to investigate the expression of eight surface and intracellular proteins in CTCs from metastatic BC patients, to assess individual response to therapy (Sinkala et al., 2017). Since therapies that target proteins are increasingly rising, monitoring protein expression in CTCs isolated from peripheral peripheral blood may guide therapeutic selection in the near future.

Recently, FC-based technologies have been used and adapted to detect multiple markers at a single cell level too. In this regard, CTCs from osteosarcoma patient blood samples were characterized by Shulin Li’s research group a couple of years ago (Batth et al., 2020). After having isolated CTCs for their positivity to vimentin and negativity to CD45, a multiplex labeling using antibodies conjugated to metal ions revealed the presence of 18 different markers simultaneously. After protein quantification, they used a tailored bioinformatic analysis to obtain unique patient-related protein signatures containing information about active signaling pathways, which could help us to predict future tumor behavior and guide treatment choice.

## Metabolomics

### Metabolomic analysis

Since the discovery of the famous Warburg effect (Warburg et al., 1927), several studies have shown that there is a broad spectrum of bioenergetic and metabolic phenotypes supporting cell proliferation, metastasis and resistance to cancer therapies. Oncometabolites are small endogenous and exogenous molecules present in tissues and biofluids, accumulated by altered metabolic pathways during malignant transformation (Khatami et al., 2019). They originate in the tumor microenvironment to create the optimal growing conditions for the tumor (Elia and Haigis, 2021; Hofer et al., 2021).

Metabolomics focuses on the profiling of small intracellular or free metabolites (≤1,500 Daltons) in bodily fluids including blood, urine, CSF and saliva (Han et al., 2021). Metabolites of interest can be detected using Nuclear Magnetic Resonance (NMR) (mostly proton NMR, H-NMR) and MS, in association with different separation methods (Fiehn, 2016; Kang et al., 2018; Lane et al., 2019; Sinclair and Dudley, 2019). Based on chemical properties of specific atoms in a molecule, NMR can be used with biological samples without prior processing, and the unaltered starting material can be re-used for additional investigations. However, compared to MS, a lower number of metabolites, with lower sensitivity (micromolar vs. nanomolar) can be identified using NMR, and for this reason MS still represents a gold standard.

### Clinical application

Metabolomics is mainly used for early cancer detection and biomarkers discovery (Schmidt et al., 2021). Uchiyama et al. showed that benzoic acid has a high diagnostic capacity in CRC and identified a correlation between CRC stages and upregulation/downregulation of different serum metabolites (Uchiyama et al., 2017). Another study based on proton NMR revealed that fecal metabolomic fingerprinting can be used as an early diagnostic tool in CC patients (Uchiyama et al., 2017). In a wide-scale metabolic investigation of plasma samples from OC patients, Ke et al. demonstrated that metabolic signatures can facilitate early diagnosis of OC, helping us to discriminate early from late stages (Ke et al., 2015).

Interesting results have been obtained from studies of metabolomic composition of urine(u)-EV of prostate cancer patients (Clos-Garcia et al., 2018). According to Puhka et al., patients before prostatectomy presented a different uEV metabolome content compared to those after prostatectomy and healthy controls (Puhka et al., 2017). A different study reported that approximately 76 metabolites were differentially expressed between patients with prostate cancer and patients with benign prostatic hyperplasia (BPH) some of which were among the metabolic alterations reported in PCa (Xu et al., 2021).

Increased evidence has demonstrated the influence of microbiota in human malignancies including cancer (ich-Poore et al., 2021). Microbiome metabolites can influence the tumor microenvironment by regulating different aspects of carcinogenesis including proliferation, angiogenesis, inflammation and metastasis (Rossi et al., 2020). The connection between serum metabolome and the intestinal microbiome in patients with lung cancer at different stages was recently investigated. As the disease progressed, the L-valine and Lachnospiraceae_UCG006reased suggesting L-valine is a potential marker for lung cancer diagnosis (Chen et al., 2022). A different research group identified specific microbiome-associated metabolites in CRC patients analyzing their fecal microbiome (Yang et al., 2019). They found proteobacteria, fusobacteria, high concentrations of polyamines (cadaverine and putrescine), amino acids (Pro, Glu) and urea in patients compared to healthy volunteers in which, on the contrary, sugars and fatty acids (Yang et al., 2019) were abundant.

In conclusion, implementation of analytical techniques and validation of algorithms for analyzing metabolomics data are still needed to let metabolomics take its rightful place among precision oncology omics.

## Bioinformatics tools for liquid biopsy

The reduced amount of target molecules in LB affects the capability to detect low-frequency genomic variations. To increase detection sensitivity, a panel of genes rather than the whole genome can be sequenced numerous times, even though this leads to higher false positivity risk. To solve this issue, advanced bioinformatic tools or machine learning algorithms are needed to reduce false positive results and eliminate background noise.

Tools such as the popular IchorCNA (Adalsteinsson et al., 2017), have been designed to estimate a low coverage plasma sample tumor fraction in an ultra LP-WGS scenario (0.1X) and help the operator decides whether enough material is available for a WES comprehensive clonal analysis. Following, in a recent paper, Zviran et al. (2020) stick to WGS and proposed replacing depth of sequencing with breath for sensitive detection of low-burden cancer, by increasing the number of detectable sites (SNVs) performing WGS at an average 35X. The WGS approach enabling effective integration across orthogonal data dimensions such as SNV and CNV allows clinical application to a wide range of tumor types that have either high mutation load or aneuploidy (Taylor et al., 2018). Unfortunately, if the technique provides sensitive detection of ctDNA it does also provide limited confidence in the sensitivity to detect any individual site so, target sequencing remains the election choice to identify driver mutational events.

Similar approaches can be used to detect CTC genome alterations. Once CTC are isolated from biological fluids their DNA is amplified and sequenced by LP or ultra LP-WGS. At a coverage of 1X or 0.1X heterogeneity that might arise due to small genomic aberrations such as SNVs and short indels will be missed. Several computational tools are available for CNV analysis using bulk sequencing data including CNVkit (Talevich et al., 2016), ControlFreec (Boeva et al., 2012), ASCAT (Van Loo et al., 2010) and Sequenza (Favero et al., 2015), just to name some, and many groups rely on these for the analysis of single cell data (Liu et al., 2019a; Pailler et al., 2019; Oulhen et al., 2021). Among the few open-source tools specifically designed for CNV calling in single cells we can list the cloud-based Ginkgo (Garvin et al., 2015) which is developed for LP-WGS data, making the CNV analysis procedure user-friendly, including for those with limited bioinformatics experience.

When the goal is to analyze large datasets, more computationally efficient strategies are required. AneuFinder (Bakker et al., 2016) and SCOPE (Wang et al., 2020) are two R-Bioconductor packages developed to explore tumor single cell data to identify evidence for copy number variations in WGS samples while among the python packages we can highlight SCNV (Wang et al., 2019), baseqCNV (Fu et al., 2019), SCCNV (Dong et al., 2020), SCICoNE (Kuipers et al., 2020) and CHISEL (Zaccaria and Raphael, 2021). If genomic alterations’ analysis is influenced by the scarcity of the starting material, investigations on RNA must be performed also taking into account its instability in biological fluids (Cheng et al., 2019; Vaisvila et al., 2021). Therefore, even if the bioinformatics pipelines currently used for CTC derived bulk and scRNAseq sequencing data are the same used for other types of transcriptomic data (Satija et al., 2015; Wolf et al., 2018) analyses of these samples require additional quality evaluation and preprocessing.

Recent research suggests that cfDNA fragmentation patterns can provide additional information beyond the genetic analysis of somatic mutations and copy-number abnormalities. Indeed, DNA fragmentation from dying tumor cells seems not randomly distributed but also determined by the DNA sequence: it appears to reflect the chromatin structure and epigenetic states of the cells, from which DNA fragments derive (Snyder et al., 2016; Cristiano et al., 2019; Sun et al., 2019). Peneder et al. (2021), in their inspiring paper, identified a specific epigenetic signature among fragmentation patterns in tumor DNA isolated from the blood of patients with Ewing Sarcoma. The authors introduced a new algorithm for detecting ctDNA based on cancer-specific chromatin signatures and combined several fragmentation-based metrics into an integrated machine-learning classifier that exploits widespread epigenetic deregulation and is tailored to cancers with few genetic lesions such as pediatric tumors.

Among the positive outcomes of multi-omic investigations there is the construction of useful databases, built to help researchers delineate which proteins are expressed in specific tissues in physiological and pathological conditions. Examples are computational resources such as the Human Protein Atlas (https://www.proteinatlas.org): a repository of information about mRNA and protein expression across several healthy tissues and cancers, and the Genotype-Tissue Expression (GTEx) project (https://gtexportal.org).

Several ML frameworks have been developed to characterize tumor biomarkers in an unbiased, automated and reproducible manner (Svensson et al., 2015; Ko et al., 2018), mostly purely based on extracted-features traditional methods. Recently, Zeune et al. (2020) showed how with a more complex, “black box” approach combining autoencoding convolutional neural networks (CNN) with advanced visualization techniques they were able to segregate 164 metastatic BC patients based on favorable and unfavorable prognosis starting from CellSearch imaging data, proving the deep learning method was at least as good as manual CTC count in cell classification. Automatic, objective cell classification with a CNN-based image processing path was followed by several other groups (He et al., 2020; Guo et al., 2022) highlighting the importance for research centers, and hospitals to work with instruments producing accessible, good quality and properly formatted CTC imaging data for reliable ML-based predictions.

## Challenges and perspectives

Although there is great potential in LB’s biomarkers, some biological and technical issues must be solved before entering into medical practice. Several completed and ongoing are going to establish whether these biomarkers can be fully adopted in clinicalisions (Table 5). However, it is not yet clear whether liquid biomarkers can capture the entire tumor heterogeneity at the time of analysis, especially in metastatic patients in which distinguishing the contribution of each site is currently not feasible. Detection and characterization of circulating tumor components, especially in the early setting, presents many difficulties due to their low amounts in biological fluids. These unresolved aspectsgenerate inconclusive and conflicting data with a high rate of false positives (overdiagnosis) and/or false negatives (underdiagnosis).

**TABLE 5:** Ongoing and completed clinical trials involving liquid biopsy in cancer management.

Trial	Biomarkers	Tumor type	Sample size	Outcome measures	Clinical application
NCT00382018 Phase 3	CTC	Breast cancer	624	Evaluation of OS and PFS in early switching to an alternative chemotherapy in CTC positive patients	Therapy selection Prognosis
NCT0180005 NA	CTC	Prostate cancer	68	Prognostic value of CTCs in prostate cancer at High Risk treated radically with radiotherapy and hormones	Prognosis Response prediction Monitoring therapy
NCT02874885 Observational	CTC	Renal cancer	520	Assess of the CTC number during neoadjuvant treatment	Response prediction
NCT05326295 Observational	CTC	Breast cancer	1,000	Evaluation of CTC number and phenotype on the prognosis of early stage BM patients treated with neoadjuvant or adjuvant chemotherapy	Prognosis Therapy monitoring
NCT04917289 Phase 3	CTC	Colon cancer	100	CTC vs. Radiography as the evidence of recurrence in CRC patients	Therapy resistance
NCT05533515 Observational	CTC	Prostate cancer	490	Establish the value of CTC positivity in predicting post-Radical Prostatectomy failure	Therapy prediction
NCT03568630 Observartional	ctDNA	Pancreatic cancer	1,250	Identification of specific ctDNA signature for early cancer detection	Early detection
NCT03664024 Phase 2	ctDNA	Non-Small Cell Lung Cancer	118	Baseline TMB assessment in ctDNA for response prediction to Pembrolizumab + chemotherapy	Therapy prediction
NCT02813928 NA	ctDNA	Colon cancer	473	Monitoring ctDNA in CRC patients after curative treatment	Diagnosis Prognosis
NCT04089631 Phase 3	ctDNA	Colon cancer	4,812	DFS and OS after Adjuvant Treatment	Therapy selection
NCT04901988 Phase 2/3	ctDNA	Melanoma	1,050	cfDNA guided therapy for stage IIB/C melanoma after surgical resection	Therapy selection
NCT05174169 Phase 2/3	ctDNA	Colon cancer	1912	Evaluation of residual disease by cfDNA for optimal adjuvant chemotherapy scheme	Minimal residual desease
NCT04629079 Observational	Exosome	Lung cancer	800	Improving the early detection of lung cancer by combining exosomal analysis of hypoxia with standard of care imaging	Early Diagnosis
NCT02702856 Observational	Exosome	Prostate cancer	2000	Correlate an exosome gene expression signature with the presence of high-grade prostate cancer	Early diagnosis
NCT05427227Observational	Exosome	Gastrointestinal cancer	500	Evaluation of proteomic profiling of tumor exosome during IC, anti-Her2 and anti-CLDN18.2 therapy	Monitoring response
NCT05705583 Observational	Exosome	Renal cell carcinoma	100	Assess the correlation between the circulating exosomes levels and the tumor responsiveness to immunotherapy	Response prediction
NCT04965259 Observational	miRNA	Hepatocellular cancinoma	2000	Evaluate changes in the profile of miRNA as high-risk patients develop HCC	Diagnosis
NCT01722851 Observational	miRNA	Breast cancer	255	Assess the relationship between changes in circulating miRNA levels and therapy response	Therapy selection Monitoring Response
NCT04771871 Phase 2	miRNA	Breast cancer	42	Evaluation of the fold change in serum levels of miRNA during standard chemotherapy	Therapy resistence
NCT05146505 Observational	miRNA	Ovarian cancer	150	Longitudinal miRNAs analysis over chemotherapy treatment	Therapeutic Monitoring
NCT02739867 Observational	TEP	Solid/hematological cancer	476	Evaluation of diagnostic accuracy of platelet RNA profiling in detecting occult cancer	Cancer screening

**FIGURE 2:**
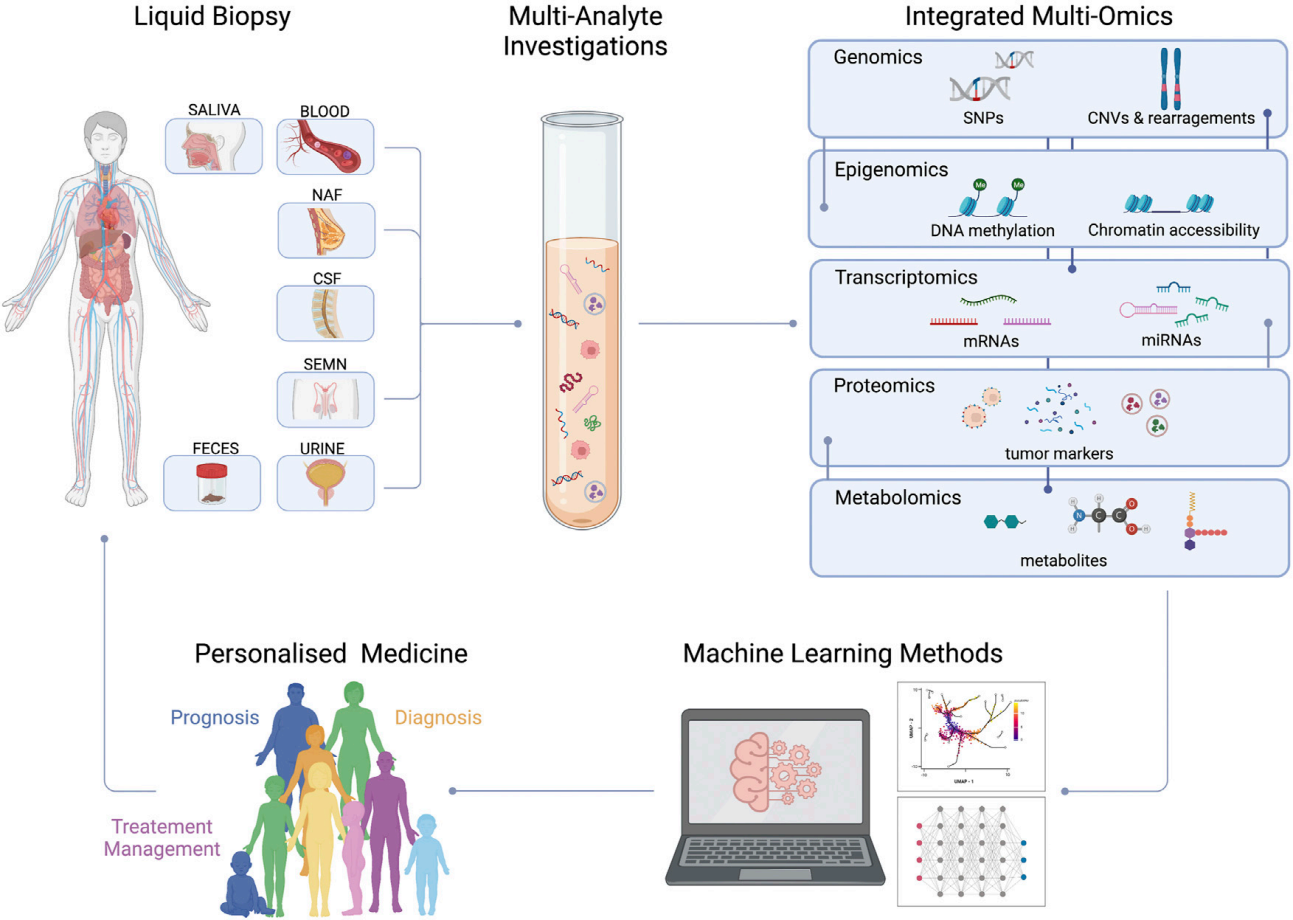
Integrated multi-omic approach in liquid biopsy. An integrated multi-omic approach is going to be used investigating single or multiple analytes from different fluids of the same patient. Artificial intelligence will be educated using the enormous amount of information that has been created. From the resulting predictive models can be originated personalized medical decisions (NAF, Nipple Aspirate Fluid; CSF,Cerebrospinal Fluid; SEMN, Seminal Fluid; CNV, Copy Number Variations; SNPs, Single Nucleotide Polymorphisms).

One possible solution that has been recently proposed is the use of a multi-omic, multi-analyte LB which could offer a high-resolution snapshot of cancer complexity (Figure 2). Indeed, the identification of a single biomarker able to reconcile biological and technical needs represents a lost cause. Clinical sensitivity and specificity of CTC and ctDNA tests can be improved when coupled with protein-based markers. First evidence is the prospective study of Imperiale et al. which compares a multitarget stool DNA test with a fecal immunochemical test (FIT) to predict the risk of developing CC. The stool test combines identification of KRAS mutation and aberrant NDRG4 and BMP3 methylation with fecal hemoglobin dosage. DNA test demonstrated to have higher sensitivity than FIT assay for both advanced precancerous lesions and colorectal cancer, although with lower specificity (Imperiale et al., 2014).

Cohen et al. developed a multi-analyte blood test, called CancerSEEK that simultaneously evaluates eight protein biomarkers and tumor-specific mutations in circulating DNA of common human cancer types (Cohen et al., 2018). The assay had a sensitivity of 69%–98% for five cancers for which no screening tests are available for high-risk individuals while specificity was greater than 99% with only 7 of 812 healthy controls testing positive. Moreover, without any clinical information on patients, the test identified the single anatomic site in a median of 63% of the patients (Cohen et al., 2018). Interesting preliminary data were obtained with a multianalyte panel based on analysis of EV RNA, cfDNA and dosage of CA19-9 protein in PDAC patients (Yang et al., 2020). Combining different blood-based biomarkers, authors distinguished patients with PDAC patients from healthy controls with 92% accuracy, 95% specificity and 88% sensitivity. This model could improve detection of occult metastases not visible with conventional imaging at baseline and only discovered intraoperatively or after 4 months of baseline blood draw (Yang et al., 2020).

On the one hand, multi omic analysis seeks to fullyipher the complexity of cancer heterogeneity, in contrast it generates a huge amount of data. Omic data can “apparently” be very different from each other as numerous biological and non-biological variables can influence their production. The choice of patients, the type of protocol adopted to perform the analysis, or the personal experience of the operator can greatly affect the final data. Over the years different repositories like TCGA (https://portal.gdc.cancer.gov/) and ICGA (https://dcc.icgc.org/) have been created to collect all omics data in an orderly manner. Since there is an urgent need to connect multi-level information concerning molecular signatures and the phenotypic manifestation of different cancer types, AI and ML approaches have been proposed to transform big-sized complex data into evidence-based medicalisions. Although there is growing evidence demonstrating the potential of ML to improve the performance of various LB tests, their integration into the clinical workflow represents the main challenge of the upcoming years (Macaulay et al., 2015).
